# Effects of neurofeedback on the self-concept of children with learning disorders

**DOI:** 10.3389/fpsyg.2023.1167961

**Published:** 2023-05-15

**Authors:** Benito Javier Martínez-Briones, Rodrigo Flores-Gallegos, Sonia Y. Cárdenas, Bertha Elena Barrera-Díaz, Thalía Fernández, Juan Silva-Pereyra

**Affiliations:** ^1^Departamento de Neurobiología Conductual y Cognitiva, Instituto de Neurobiología, Universidad Nacional Autónoma de México Campus Juriquilla, Querétaro, Mexico; ^2^Universidad Pedagógica Nacional, Querétaro, Mexico; ^3^Facultad de Estudios Superiores Iztacala, Universidad Nacional Autónoma de México, Tlanepantla, Estado de México, Mexico

**Keywords:** self-concept, self-esteem, neurofeedback, learning disorder, children, EEG biofeedback

## Abstract

Children with learning disorders (LDs) often have a lower self-concept than their typically developing peers. Neurofeedback (NFB) treatments seem to improve the cognitive and academic performance of these children, but the effects on self-concept have not been studied. In this exploratory study, 34 right-handed children (8–11 y.o.) with LD and delayed electroencephalographic maturation responded to the Piers–Harris Children’s Self-Concept Scale. One group received NFB (*n* = 20), and another group (*n* = 14) served as control, which included 9 children treated with sham-NFB and 5 on a waiting-list. A nonparametric permutation approach was used to compare the academic performance and self-concept difference (postscores – prescores) between the NFB and control groups. Given the smaller size of the control subgroups, a comparison of the percent changes between sham-NFB and the waiting-list was performed with the non-overlap of all pairs (NAP) technique. In the NFB group, the scores of reading, math, and global self-concept increased significantly, highlighting the self-concept subdomains of physical appearance, nonanxiety, popularity, and happiness. Additionally, the sham-NFB subgroup showed better outcomes than the waiting-list subgroup, perhaps due to noncontrolled factors. We found improved academic performance and self-concept in children with LDs who received NFB treatment. This study is an important exploratory step in studying a relevant treatment that seems to ameliorate symptoms of LDs such as anxiety and low self-concept.

## 1. Introduction

With a prevalence range of 5–20%, learning disorders (LDs) are the most common neurodevelopmental problems afflicting school-age children (Shaywitz et al., 1999; Altarac and Saroha, 2007; Lagae, 2008; American Psychiatric Association, 2013). According to the Diagnostic and Statistical Manual of Mental Disorders, Fifth Edition, Text Revision (DSM-5-TR) (American Psychiatric Association, 2022), a specific LD is diagnosed in individuals with persistent difficulties (at least 6 months) during their development (Criterion C) in learning the basic academic skills of reading, writing, or mathematics (Criterion A), with performance scores in standardized tests substantially below those expected for their age, causing significant interference with academic performance or with activities of daily living (criterion B). The learning difficulties are not better explained by intellectual disabilities, uncorrected visual or hearing acuity, other neurological or psychiatric disorders, or inadequate educational instruction (Criterion D).

Compared with children with typical development, students with LD have higher rates of emotional disorders such as anxiety, depression (Willcutt and Pennington, 2000; Carroll et al., 2005; Nelson and Gregg, 2012), and an affected sense of self-esteem or self-concept (both concepts being often conflated; Cooley and Ayres, 1988; Smith and Nagle, 1995; Gans et al., 2003; McArthur et al., 2016, 2020; Huang et al., 2021). Self-esteem is a rather general and emotionally loaded value that people assign to themselves, while self-concept is a psychological construct of how people perceive themselves based on a multifaceted set of relatively stable self-perceptions, formed through experience and influenced by the judgments of others, which includes a sense of social worth and thoughts about one’s physical characteristics, abilities, and academic skills (Epstein, 1973; Marsh, 1990; Piers and Herzberg, 2002; Zeleke, 2004; McArthur et al., 2020). Self-concept is “essentially phenomenological in nature”; therefore, it heavily depends on the self-report of the individual to describe and evaluate him or herself (Marsh, 1990; Piers and Herzberg, 2002). Since school is considered the main social environment for young people, individuals who are receiving failing grades are more at risk of developing negative self-concepts, anxiety, and depression. Reduced self-esteem is in itself an important risk factor for depression in the young (Sowislo and Orth, 2013; Choi et al., 2019; Hards et al., 2020); it has a bidirectional relationship with anxiety (Sowislo and Orth, 2013; Francis et al., 2019), and teens with LDs show three times more suicidal ideations and attempted suicides than their peers (Daniel et al., 2006). LDs have significant societal impacts in the form of school dropout and higher levels of poverty, with most juvenile delinquents showing low academic performance (Kutner et al., 2006); thus, it is important to explore the emotional and identity dimensions of individuals with LDs and the impact of treatments to ameliorate their symptoms.

The current research on self-concept impairments in LDs either focuses on heterogeneous samples of academic impairments, mostly working with the formerly known learning disorder not otherwise specified (LD-NOS) (American Psychiatric Association, 2000), or on the dyslexia subtype (Snowling et al., 2007; Terras et al., 2009; Huang et al., 2021), with meta-analyses showing people with dyslexia (compared to controls) having an affected sense of global self-concept together with an impaired self-perception of academic skills (McArthur et al., 2016) and anxiety (Francis et al., 2019; McArthur et al., 2020). In samples with heterogeneous types of LDs, self-esteem has been found to be affected (Lahane et al., 2013), with specific impairments in the self-concept subdomains of academic skills and conduct (Cooley and Ayres, 1988; Gans et al., 2003), including affected perceptions of intellectual ability and social acceptance (Smith and Nagle, 1995).

The main interventions to treat the academic symptoms of LDs are special education classes and remedial programs in reading, writing, or mathematics (Swanson and Hoskyn, 1998; National Reading Panel, 2000; Shaywitz and Shaywitz, 2020). It is assumed that a child’s self-concept would be indirectly improved by successfully treating their main academic impairments, due to supporting their ability to perform at school, coupled with the positive feedback from their achievements and encouragement from others (McArthur et al., 2016). However, it is not common practice to report self-concept improvements, or even general improvements in well-being, in treatments that focus on academic domains, but two studies do stand out. On the one hand, Block (1993) reported improved self-esteem together with reading improvements in children who went through a literature-based reading program; on the other hand, a meta-analysis of educational interventions found a moderate effect of treatments on the self-concept of children with LDs (Swanson and Hoskyn, 1998).

Regarding the effects of noneducational types of treatment on the self-concept of people with LDs, MacMahon and Gross (1987) treated LD children with an aerobic exercise program and found that their self-concept improved compared to a control group. Similarly, Musetti et al. (2019) found that teenagers with LDs who underwent psychosocial treatment improved their self-concept compared with healthy or untreated teens with LDs.

An EEG-based neurofeedback (NFB) treatment is also a relevant therapeutic approach. An NFB treatment is an operant conditioning training program that aims to modify brain activity for therapeutic or performance-enhancing purposes (Budzynski et al., 2009; Gruzelier, 2014; Sitaram et al., 2017). NFB treatments have an experimental treatment status (Thibault and Raz, 2016), with ongoing research of their effects on disorders such as ADHD (Lubar et al., 1995; Simkin et al., 2014, 2016), anxiety disorders (Hammond, 2006; Abdian et al., 2021), epilepsy (Egner and Sterman, 2006; Sterman and Egner, 2006; Morales-Quezada et al., 2019), and LDs (Fernández et al., 2003; Becerra et al., 2006; Breteler et al., 2010; Nazari et al., 2012; Martínez-Briones et al., 2021). Children with LD often exhibit an abnormally slower resting-state EEG than children with typical development, characterized by an excess of theta activity and a deficit of alpha activity (Chabot, 2001; Fernández et al., 2002; Fonseca et al., 2006). The research of NFB effects on LD shows that attempting to normalize the EEG by reducing the theta/alpha ratio seems to facilitate EEG maturation and, as a consequence, can boost cognitive performance (Fernández et al., 2003, 2016; Martínez-Briones et al., 2021) and improve EEG resting-state patterns (Fernández et al., 2003, 2007), with treatment effects lasting at least 2 years (Becerra et al., 2006). NFB treatments may also benefit those with LDs by improving spelling ability, which may be associated with increased EEG connectivity of the alpha-band (Breteler et al., 2010), and by improving reading and phonological awareness, with such effects possibly being related to the normalization of EEG connectivity measures (Nazari et al., 2012). However, to our knowledge, there is no evidence of an improved self-concept after NFB treatment in LD children. Three of the abovementioned studies state that most parents subjectively reported a boost in their child’s self-esteem (Becerra et al., 2006; Fernández et al., 2007, 2016), but this was not captured with a direct and objective assessment that considered self-concept as a multidomain construct. Thus, this study aimed to explore the effects of NFB treatments on the self-concept of children with LDs.

The LD sample of this study was heterogeneous, with impairments in the academic domains of reading, writing, and/or mathematics. We adhered to an examination of a global self-concept derived from the following subdomains or specific perceptions of self-concept: behavior, intellectual or academic skills, physical appearance, freedom from anxiety, popularity, and happiness or life satisfaction (Alexopoulos and Foudoulaki, 2002; Flahive et al., 2015). Hence, this is an exploratory study of the possible effects of NFB treatment on six aspects of self-concept in children with LDs.

Several researchers conceive cognitive achievement as the result of self-esteem or self-concept (Marsh, 1990; Núñez Pérez and Solís, 1995; González-Pienda et al., 2000; Tobia et al., 2017); however, Baumeister et al. (2003) views a high self-concept as partly the result of good school performance. In a meta-analysis based on the analysis of 105 studies involving a sample of more than 58,000 participants from the world over, Huang (2011) concluded the relationship between self-concept and academic performance as bidirectional. Since the children in this study come from primary schools, it is essential to recognize the relationship between academic performance and self-concept as stronger in elementary than in high school (Huang, 2011). Thus, because the NFB treatment aims to improve cognitive performance, an increased self-concept may be an emergent result of this study.

## 2. Materials and methods

The Ethics Committee of the Instituto de Neurobiología, Universidad Nacional Autónoma de México (UNAM), approved the experimental protocol (INEU/SA/CB/146). This protocol complies with the Ethical Principles for Medical Research Involving Human Subjects established by the Declaration of Helsinki (World Medical Association, 2013). Informed consent was signed by all children and their parents.

### 2.1. Participants

The necessary sample size for this study was calculated with G*Power 3.1 software^1^ using the effect size of a difference between two groups (NFB vs. sham-NFB; Martínez-Briones et al., 2021). We used the following values: a Cohen’s *d* effect size of 1.15, a 1:1 size ratio between the two groups, a one-tailed type 1 error rate of 0.05, and a power of 0.9. Accordingly, at least 28 participants (14 per group) were needed.

Forty right-handed children aged 8–11 years diagnosed with LD were selected from a larger sample of children referred by teachers and social workers from several elementary schools in Querétaro, México. All children fulfilled the following inclusion criteria: (1) a normal neurological and psychiatric assessment (except for the LD diagnostic requirements, as stated below), without language impairments or visual/ hearing acuity problems (those with visual problems used correcting glasses); (2) an intelligence quotient (IQ) of at least 75 [Wechsler Intelligence Scale for Children 4th Edition, WISC-4 (Wechsler, 2010)], used to exclude children with intellectual disability; (3) without severe socioeconomic disadvantages, that is, a mother (or tutor in her absence) with at least a completed elementary school education and a *per capita* income greater than 50 percent of the minimum wage; and (4) an abnormally high EEG resting-state theta/alpha ratio compared to a normative database (Bosch-Bayard et al., 2020b). The EEG of children with LDs often has more theta and less alpha activity than typical children; thus, we obtained the *z* values of the theta/alpha ratio and selected children with *z* values greater than 1.645 (one-tailed distribution, *p* = 0.05) in at least one lead of their EEG spectra.

In addition, all children had an LD diagnosis. The LD diagnosis was based on the following three criteria: (a) poor academic achievement reported by teachers and parents; (b) percentiles of 10 or lower in the subscales of reading, writing, or mathematics of the Infant Neuropsychological Scale for Children (Matute et al., 2014); and (c) the final decision of LD was delivered by a psychologist according to the DSM-5 criteria for LD (American Psychiatric Association, 2013). A few children failed to complete different items of the attentional evaluation, but they did not meet the DSM-5 criteria of ADHD (American Psychiatric Association, 2013), as others have reported (Holcomb et al., 1986; Silva-Pereyra et al., 2003).

All children were randomly assigned to either an NFB treatment that reinforced a reduction in the theta/alpha ratio (NFB group) or a sham-NFB treatment (control group). The treatment (NFB or sham) was delivered via the lead with the highest abnormal *z* value.

**Figure 1:**
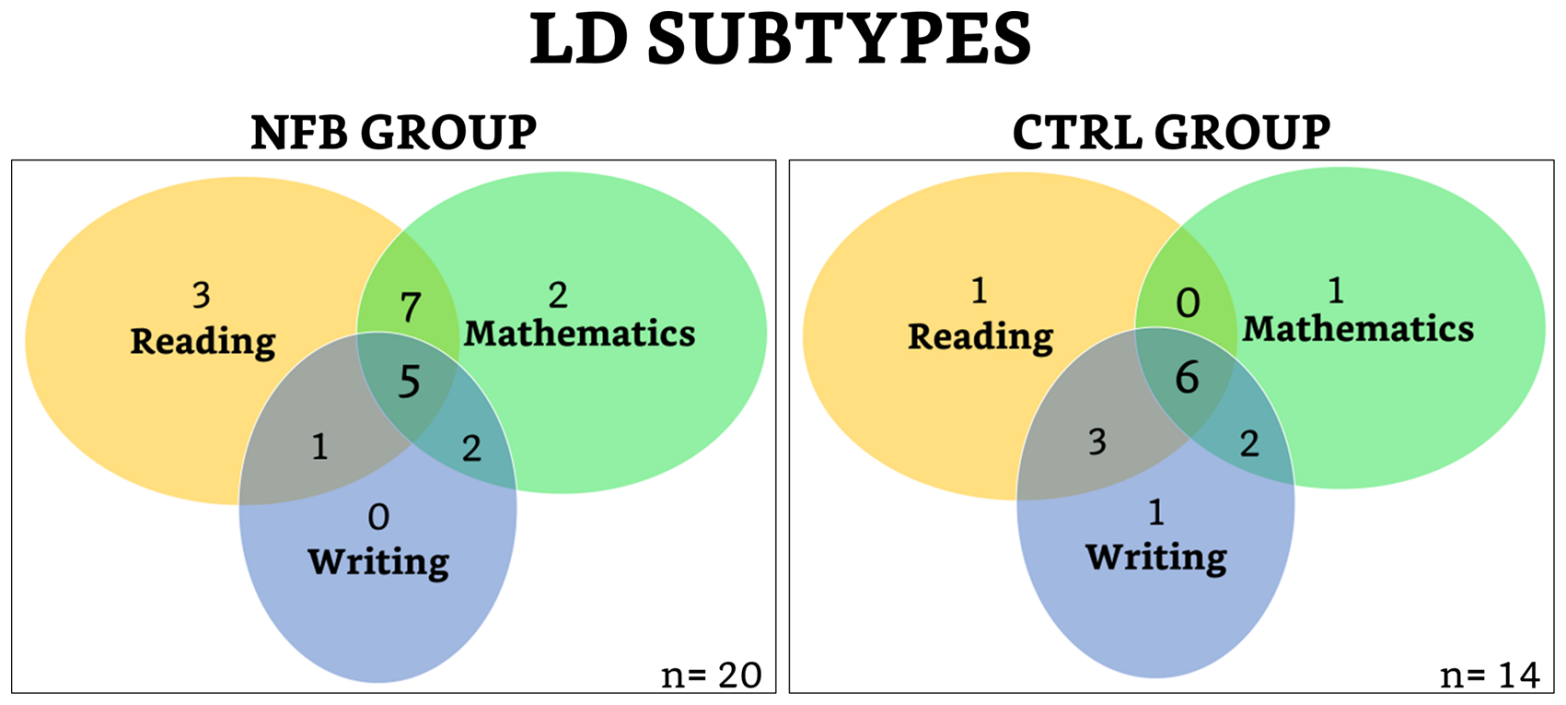
Venn diagrams of the distribution of academic impairments.

Eleven children were impaired in all three domains (reading, writing, and mathematics); four children were impaired in reading and writing; seven children were impaired in reading and mathematics; four children were impaired in writing and mathematics; four children were impaired in reading; one child was impaired in writing; and three children were impaired in mathematics (Figure 1). It can be noted that our sample of children with LDs was heterogeneous in its distribution of academic impairments, yet both groups were reasonably similar (see Table 1 and Figure 1) for comparison in further analyses.

**Table 1:** Descriptive data for the neurofeedback (NFB) and control (CTRL) groups.

	NFB *n* = 20	CTRL *n* = 14	Statistical differences between groups	
	Mean (SD)	Mean (SD)	*t*	*p*
Age	9.05 (1.05)	9.00 (1.52)	0.11	0.91
Female/male ratio	9/11	6/8	*X*^2^ = 0.15	0.90
WISC-4: Full Scale IQ	92.55 (11.06)	93.07 (9.14)	−0.15	0.89
Reading	30.62 (20.87)	21.16(20.48)	1.36	0.26
Writing	38.68 (21.07)	28.82(17.80)	1.52	0.20
Mathematics	32.57 (20.79)	41.01(21.78)	−1.17	0.31
Global self-concept	53.15 (11.90)	59.79(8.36)	−1.96	0.04
z score (theta/alpha)	2.62 (1.001)	2.19(0.57)	1.22	0.18

All children were randomly assigned to either an NFB treatment that reinforced a reduction in the theta/alpha ratio (NFB group; *n* = 20, 9 females) or a sham-NFB treatment (control group). The treatment (NFB or sham) was delivered via the lead with the highest abnormal z value. However, only 9 children in the control group received sham-NFB treatment; the remaining 11 could not receive any treatment due to COVID-19 pandemic-related lockdowns. Nonetheless, we were able to carry out the evaluations for 5 of them after a period of waiting. Therefore, the control group (Ctrl group; *n* = 14, 6 female) was made up of two subgroups of children: a sham subgroup (*n* = 9) and another subgroup, which we considered to be a waiting-list group (WL group; *n* = 5).

### 2.2. Instruments

#### 2.2.1. Neuropsychological scale for children (ENI)

The Neuropsychological Scale for Children (ENI: Escala Neuropsicológica Infantil; Matute et al., 2014) is standardized by age for the Mexican population. Three ENI domains are evaluated, namely, reading, writing, and mathematics, with several variables assessed for each domain (reading: accuracy, comprehension, and speed; writing: accuracy, narrative composition, and speed; mathematics: counting, number management, calculus, and logical reasoning). Raw scores were transformed to percentiles according to the scale attributes. Reading and writing speeds were measured in terms of the time needed to read a text and write a composition; the correct responses were measured for the other subdomains of the three ENI domains.

#### 2.2.2. Piers–Harris children’s self-concept scale

All participants responded to the Piers–Harris Children’s Self-Concept Scale, a self-report questionnaire chosen for its multidimensional structure, which allows the categorization of self-perceptions of different domains of experience. The scale’s items describe real scenarios with which the children could feel identified. Participants were instructed to answer yes or no to a list of 80 statements about how they think and feel about themselves. A psychologist clarified the confidentiality of the test and explained the importance of giving truthful answers. The psychologist was also present during the performance of the scale to assist with children’s doubts.

The scale gives a global score as a general measure of self-concept taken from 6 specific subdomains: behavior, academic competence, physical appearance, freedom from anxiety, popularity, and happiness. A higher score indicates a more positive self-evaluation in the measured subdomain. In this study, the Piers–Harris 2 was used. It was standardized by scholastic grade with a U.S. sample of 1,387 children (49.7% male and 50.3% female) ranging from 7 to 18 years. We considered that the sample may be slightly underrepresentative of a Hispanic or Latino population; however, it has been recognized as appropriate in research, educational, and clinical settings (Alexopoulos and Foudoulaki, 2002; Review of the Piers-Harris Children’s Self-Concept Scale 2nd Edition, 2011; Flahive et al., 2015).

### 2.3. Neurofeedback and sham treatments

A resting-state EEG was recorded during an eyes-closed condition while the child was seated in a dimly lit, faradized, and soundproofed room using 19 leads of the 10–20 International System (ElectroCap™ Inc., Eaton, OH, United States) referenced to linked earlobes (A1A2). For this purpose, we used a Medicid™ IV system and Track Walker™ v2.0 software (Neuronic Mexicana, SA, Mexico City, Mexico). The amplifier bandwidth was set from 0.5 to 50 Hz. All electrode impedances were a maximum of 10 kΩ, and the signal was amplified with a gain of 20,000. EEG data were sampled with a frequency of 200 Hz and edited offline. On average, 24 artifact-free segments of 2.56 s were used for analysis.

To obtain the theta(θ)/alpha(α) (θ/α) ratio, first, the absolute power (AP) of the broad-band model was calculated in the frequency domain, and then θ/α was obtained as the ratio of AP(θ) to AP(α) for each lead. Here, we used the theta and alpha frequency bands in their traditional definitions: theta comprises the frequencies of 3.6–7.5 Hz, and alpha comprises the frequencies of 7.6–12.5 Hz (Fernández et al., 2003), with a frequency resolution of 0.39 Hz.

To calculate the *z* value of the theta/alpha ratio (z[θ/α]), we obtained the population age-dependent mean [μ(age)] and standard deviation (σ) for the eyes-closed resting-state EEG for each lead used in our study. This was performed by calculating the θ/α index in each lead for all subjects of the Cuban normative database (Bosch-Bayard et al., 2020b) and 2nd-order polynomial age-dependent regressions of those indices to obtain μ(age) and σ (Bosch-Bayard et al., 2001, 2020a).

The NFB treatment was applied at the lead with the highest *z*(θ/α) using a neurofeedback program adapted by Fernández et al. (2003) for the Medicid IV recording system. Every 20 ms, this program automatically selects a 1,280 ms segment and calculates the θ/α ratio. This ratio is compared to the threshold value previously established by the therapist; only if the θ/α ratio is lower than the threshold value is a tone of 500 Hz at 60 dB (positive reinforcer) emitted. This process is repeated until the EEG recording finishes, using overlapped 1,280 ms segments. The child is told to keep the sound going because it means their brain is working well; in this way, the tone assumes a positive value. The criterion for establishing this threshold the first time was using the subject’s value in their resting-state EEG recorded in the sample selection phase, but this was adjusted by trial and error until the tone was delivered approximately 70% of the time. Later (every 3 min), it was verified whether the percentage of time remained between 60 and 80% of the 3 min period, and if so, the threshold was not modified further. If the tone appeared for more than 80% of the time, the most common situation, the therapist changed the threshold to a lower value. Likewise, if the tone appeared less than 60% of the time, the threshold was increased.

The sham treatment was identical to the NFB treatment, except that it was noncontingent with the EEG activity of the child. The goal of a sham-NFB treatment is that the individual has the “feeling” of receiving a real treatment; for this, the same rewarding stimulus of the real NFB is given, but this is not related to their brain activity. There are several ways to obtain this kind of fake stimulus: one is by using the stimulus produced by recording a real NFB treatment of another participant, and the other is by randomly emitting the stimulus with a given frequency, such as between 60 and 80% of the time. The latter approach was used in this study. In other studies, some participants who received the sham treatment reported “finding the feedback confusing and ineffective” (Angelakis et al., 2007); no child in our sham group reported anything of that nature. In this study, none of the participants knew which condition they were in, nor did they know that there were both experimental and control conditions.

Each subject received 30 training sessions three times a week over 10–12 weeks, with a duration of 30 min per session. At the beginning of each session, the children were told that they would receive candy at the end of the session according to their performance. To motivate the child, a learning curve plot was updated for each session showing the last successful θ/α ratio.

All children were examined with the ENI and self-concept scales in both pre- and posttreatment conditions.

### 2.4. Data analysis

#### 2.4.1. Pretreatment comparison between groups

A non-parametric permutation *t-*test (5,000 permutations) was applied for the comparison between groups in terms of age, *z* score of the theta/alpha ratio, academic performance (reading, writing, and mathematics), and global self-concept using a statistical tool from eLORETA software (Pascual-Marqui et al., 2011). This nonparametric technique does not require a theoretical distribution since the null-hypothesis distribution of statistical tests is iteratively generated by shuffling processing of the data and does not need corrections for multiple comparisons when several time points are assessed. A chi-square analysis was performed to assess whether the sex distribution was homogenous between groups using SPSS (version 25).

#### 2.4.2. Pre- vs. posttreatment comparison between and within groups

*Z* score of theta/alpha ratio after treatment: A non-parametric permutation t test (5,000 permutations) was performed to compare the difference (postscores – prescores) between the NFB group and the sham subgroup. This analysis was not applied to the waiting-list subgroup, given its lack of postevaluation.

Academic performance after treatment: The academic performance was analyzed using the same permutation t test described before to compare the NFB and Ctrl groups’ percentile score differences (postscores – prescores) in the reading, writing, and mathematics domains. A similar analysis was applied to observe the differences within groups.

Self-concept after treatment: The permutation t test was performed to compare the differences (postscores – prescores) between the NFB and Ctrl groups in the global self-concept score. The same statistical analysis was applied to the self-concept subdomains (behavior, academic skills, physical appearance, non-anxiety, popularity, and happiness).

#### 2.4.3. Sham vs. waiting-list

Given that the Ctrl group consisted of two subgroups (sham and waiting-list), we were interested in analyzing possible between-group prepost changes in academic performance and global self-concept. Due to their small sample size, a qualitative comparison based on the percent changes was performed with the nonoverlapping all pairs (NAP) technique (Parker and Vannest, 2009) using the web-based NAP calculator from Vannest et al. (2016). With this, each variable of the pre- and postconditions was computed from the scaled scores of the respective subscales of each variable. For example, reading depends on reading accuracy, reading comprehension, and reading speed, while global self-concept depends on the following subdomains: behavior, academic skills, physical appearance, nonanxiety, popularity, and happiness. A similar description of the technique is given by Parker et al. (2011) and Flores-Gallegos et al. (2022).

## 3. Results

### 3.1. Pretreatment comparison between groups

In the comparison between the NFB and Ctrl groups, no significant differences were found in age, gender distribution, intelligence coefficient (IQ), academic performance, or z scores of theta/alpha ratio. The Ctrl group had a higher global self-concept than the NFB group, as shown in Table 1.

### 3.2. Pre vs. posttreatment comparison between and within groups

#### 3.2.1. Theta/alpha ratio

There was no significant difference between the NFB group (mean difference = −0.64, SD = 1.05) and the sham subgroup (mean difference = −0.45, SD = 0.45) in the theta/alpha ratio change (postscores – prescores) (*t* = −0.52, *p* = 0.34, *d* = −0.24). The within-group analyses showed a significant decrease in the theta/alpha ratio after treatment for both the NFB group and the sham subgroup (Table 2).

**Table 2:** Within groups pre vs. post *z* score (theta/alpha) differences for NFB and sham.

	*n*	Mean pre (SD)	Mean post (SD)	*t*	*p*	Cohen’s *d*
NFB	20	2.62 (1.01)	1.98 (1.16)	−2.70	0.00	0.58
Sham	9	2.19 (0.57)	1.74 (0.51)	−2.98	0.01	0.83

#### 3.2.2. Academic performance

**Figure 2:**
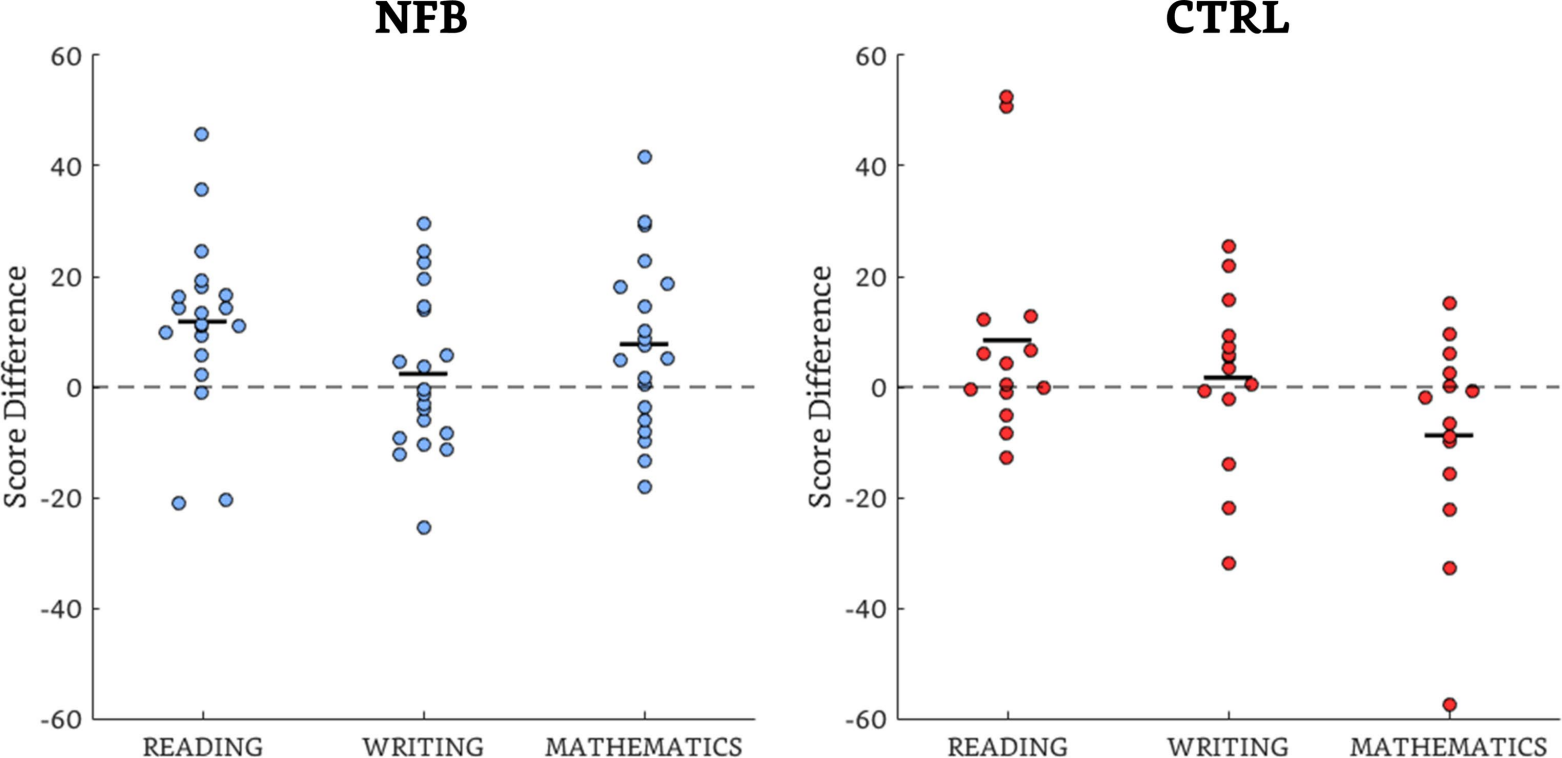
Scatterplots of the percentile difference scores (post – pretreatment)of the academic performance for the NFB and Ctrl groups. The majority of observations are above the unity (dotted) line, showing overall group effects in all but the writing domain of the NFB group and the mathematics domain of the Ctrl group. The black dashes indicate the mean values.

There was a significant gain for the NFB group (mean difference = 7.74, SD = 15.75) in mathematics (*t* = 2.86, *p* = 0.01, *d* = 0.80) compared to the Ctrl group (mean difference = −8.78, SD = 18.89), with 14/20 subjects of the NFB group improving compared to 5/14 controls, as Figure 2 shows. The within-group analyses showed a significant improvement in reading for the NFB group (*t* = 3.46, *p* = 0.005, *d* = 0.59), with 17/20 subjects improving, while there were no significant differences posttreatment for the Ctrl group (Figure 2B). Additional data can be found in the Supplementary Tables S1–S3.

#### 3.2.3. Self-concept

There was a significant gain for the NFB group (mean difference = 7.90, SD = 7.91) in the global self-concept difference (postscore – prescore) (*t* = 1.69, *p* = 0.05, *d* = 0.59) in comparison to the Ctrl group (mean difference = 2.14, SD = 11.48), with 17/20 subjects improving in the former group compared to 9/14 in the latter (including 3/5 subjects in the waiting-list subgroup).

**Figure 3:**
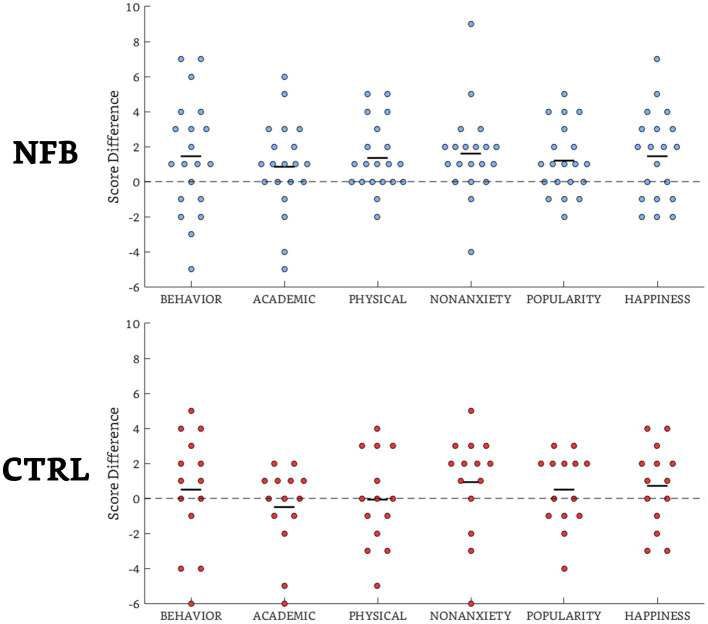
Scatterplots of the difference scores (post-pretreatment) of the self-concept subdomains for the NFB group and Ctrl groups. The majority of observations are above the unity (dotted) line, showing overall group effects in all but the subdomains of academic skills and physical appearance of the Ctrl group. The black dashes indicate the mean values.

The within-group analysis (Figure 3) indicated significant increases (*p* < 0.05) in self-concept for the NFB group in the following subdomains: physical appearance (12/20 subjects), nonanxiety (15/20), popularity (12/20), and happiness (12/20). There were no significant differences for the Ctrl group. Additional data can also be found in the Supplementary Tables S4, S5.

### 3.3. Sham vs. waiting-list

In the comparison between the sham and waiting-list subgroups, there were no significant differences in the gender distribution (*X*^2^ = 0.93, *p* = 0.33) or intellectual coefficient (IQ, *t* = −1.23, *p* = 0.27) before treatment. There was a significant difference in age between subgroups (*t* = 5.81, *p* = 0.001), but this did not affect the qualitative analysis.

The sham subgroup had a higher percent change in NAP value over the waiting-list subgroup in the academic performance domains of reading, writing, and mathematics, as shown in Table 3.

**Table 3:** Non-overlap of all pairs (NAP) assessment of academic performance for sham and waiting-list subgroups.

Variable	Group	NAP average (SD)	z	Confidence Interval 90%	Above 50% of NAP
Reading	Sham	0.50 (0.06)	7.96	0.40–0.61	66.67%
	WL	0.50 (0.08)	5.86	0.36–0.64	60.00%
Writing	Sham	0.44 (0.08)	5.21	0.30–0.58	22.22%
	WL	0.41 (0.11)	3.65	0.23–0.60	20.00%
Mathematics	Sham	0.43 (0.09)	4.85	0.28–0.57	44.44%
	WL	0.23 (0.12)	2.07	0.05–0.44	0.00%

**Figure 4:**
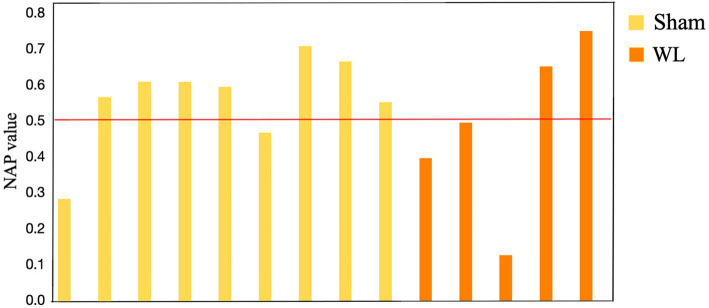
Non-overlap of all pairs (NAP) assessment of global self-concept for participants in the sham subgroup (7/9 above 0.5 NAP) and waiting-list subgroup (2/5 above 0.5 NAP).

There was also a higher NAP value for global self-concept in the sham subgroup (NAP = 0.55, SD = 0.12, *z* = 4.76, CI 90% [0.36–0.74]) compared to the waiting-list subgroup (NAP = 0.48, SD = 0.16, *z* = 3.06, CI 90% [0.22–0.73]), as shown in Figure 4.

## 4. Discussion

The main objective of this study was to explore the indirect effects of NFB on the self-concept of children with LDs. We expected the children in the NFB group to show an improved self-concept compared to the Ctrl group. Additionally, since it has been reported that NFB treatments have a direct effect on the EEG activity of LD children (Fernández et al., 2003), with a concomitant positive impact on academic performance (Breteler et al., 2010; Nazari et al., 2012), we also expected the NFB group to show a larger theta/alpha ratio decrease and better academic performance. As discussed below, these expectations were mostly met.

Regarding the changes produced by each respective treatment in the theta/alpha ratio, no significant differences were found between the NFB and sham groups. However, the within-group analyses did show significant ratio reductions for both groups. In the NFB group, this was an expected and desired result, since this reduction is an index of the operant learning involved in NFB treatments. For the sham group, some reduction in any case would be anticipated due to expectation (Schönenberg et al., 2021), the placebo effect (Geuter et al., 2017), and meta-cognitive mechanisms (Huang et al., 2020). However, above all, since the comparison was made between the NFB group and the sham subgroup (9 children) of the Ctrl group, the statistical power could not have been optimal to detect a proper difference due to the sample size.

Regarding the academic performance comparison, our heterogeneous sample of children with LDs was mainly affected in terms of reading and mathematics abilities; a corresponding improvement was observed only for the NFB group and only in those domains. A boost in reading ability has been previously found in children with LDs after receiving an NFB treatment (Breteler et al., 2010; Nazari et al., 2012), but, to our knowledge, the present study is the first that also reported an improvement in mathematics after an NFB treatment.

Concerning the main self-concept results, the impaired domains reported in the field of dyslexia are the global self-concept and the subdomains of academic skills (McArthur et al., 2016) and anxiety (Francis et al., 2019; McArthur et al., 2020). In heterogeneous LD populations such as ours, specific impairments have been found in the subdomains of academic skills, behavior (Cooley and Ayres, 1988; Gans et al., 2003), intellectual ability, and social acceptance (Smith and Nagle, 1995). Thus, after the NFB treatment, we would expect improvements in the self-concept perceptions of academic skills, behavior, and popularity (as an index of social acceptance) on the Piers–Harris scale. In this study, we found that the NFB group showed an improved global self-concept, highlighting improvements in the following subdomains: physical appearance, nonanxiety, popularity, and happiness. It has been reported that NFB treatments can positively affect anxiety (Mennella et al., 2017; López-Pinar et al., 2020; Chen et al., 2021) and depression (Choi et al., 2010; López-Pinar et al., 2020), findings that are congruent with our increased self-concept perceptions of non-anxiety and happiness. In the Piers–Harris scale, the global self-concept directly depends on the significantly affected subdomains. According to our results, the nonanxiety dimension had the largest effect size and may have exerted the most influence over the other subdomains. These results are congruent with previous findings of a prominent role of anxiety and its possible bidirectional relationship with self-concept (Sowislo and Orth, 2013).

Although the sample sizes of the Ctrl subgroups differed, the NAP analysis over each participant represented a percent change that reduced the limitation of the low statistical power. This analysis revealed that the academic performance and global self-concept in the sham subgroup improved more than in the waiting-list subgroup, which indicates a treatment effect that may be due to placebo. The placebo effect of a sham-NFB treatment arises from the technological context (e.g., noticing the signals from the EEG in a computer) and the encouragement and verbal information from the researchers and parents around the training sessions (Colloca and Miller, 2011; Schönenberg et al., 2021), which are factors that, by causing an expectation of improvement, end in a placebo response in the person. Moreover, the placebo effect could be linked to endorphin and dopamine increases that may affect the EEG alpha activity (Thornton, 2018), a finding that could also explain the lack of difference between the NFB and sham’s theta/alpha ratio change, with both groups showing a significant ratio decrease. In the sham subgroup, the placebo effect seemed to have affected the academic performance and global self-concept, with larger improvements above the waiting-list subgroup. A similar finding was reported by Qiu et al. (2022), where a placebo group with explicitly detailed information about the self-esteem and fitness benefits of a physical training session was compared to a simpler placebo group (with less explicit information) and a control group, with the placebo group improving over the control group but not as much as the explicit placebo. Thus, a placebo effect would explain some of the academic performance and self-concept improvements in our groups.

Although the previous analysis indicated that 14 individuals per group were required for a statistical power of 0.9 with an error rate of 0.05, we realize that the sample sizes are small and make generalization difficult. However, these recent times of transition are not ideal for increasing sample sizes or conducting new experiments that include children with LD, as the diagnosis is based on pre-pandemic norms, and this period was characterized by poor school instruction (Lewis et al., 2021). Also, social isolation had negative psychological effects, promoting anxiety, depression, and other factors that could affect self-concept (Orgilés et al., 2020; Xie et al., 2020).

## 5. Conclusion

Neurofeedback treatments have previously been used to ameliorate the academic impairments of children with LDs. Since a child’s self-concept might be indirectly improved by treating such impairments, this is the first exploratory study that aimed to investigate the effects of NFB on the self-concept of children with LDs. We found a positive effect of NFB on the global self-concept of these children, possibly due to the improved perceptions of physical appearance, nonanxiety, popularity, and happiness. Future studies could attempt to replicate these findings with a larger sample of children with LDs and delayed EEG maturation.

## Data availability statement

The datasets presented in this study can be found in online repositories. The names of the repository/repositories and accession number(s) can be found at: https://data.mendeley.com/datasets/s3dtfw96cx.

## Ethics statement

The studies involving human participants were reviewed and approved by Comité de Ética del Instituto de Neurobiología, UNAM. Written informed consent to participate in this study was provided by the participants’ legal guardian/next of kin.

## Author contributions

SC and TF: conceptualization. BM-B, RF-G, and JS-P: methodology and formal analysis. BM-B and RF-G: software, writing – original draft preparation, and visualization. BM-B, RF-G, SC, BB-D, and TF: investigation. BM-B, RF-G, and TF: resources. BM-B, RF-G, SC, and BB-D: data curation. BM-B, RF-G, SC, BB-D, TF, and JS-P: writing – review and editing. TF and JS-P: supervision and project administration. All authors contributed to the article and approved the submitted version.

## Funding

This research was supported by CONACYT under the grant CB-2015-1-251309, and by grant IN207520 from PAPIIT, DGAPA-UNAM, Mexico. BM-B is thankful for a CONACYT scholarship (477474) and a DGAPA-UNAM scholarship (B22298), which supported the realization of this work. RF-G (scholarship recipient: 917148) was the beneficiary of a CONACYT scholarship (720424).

## Conflict of interest

The authors declare that the research was conducted in the absence of any commercial or financial relationships that could be construed as a potential conflict of interest.

## Publisher’s note

All claims expressed in this article are solely those of the authors and do not necessarily represent those of their affiliated organizations, or those of the publisher, the editors and the reviewers. Any product that may be evaluated in this article, or claim that may be made by its manufacturer, is not guaranteed or endorsed by the publisher.

## Footnotes

^1^ https://www.psychologie.hhu.de/arbeitsgruppen/allgemeine-psychologie-und-arbeitspsychologie/gpower

## References

[ref1] Abdian H. Rezaei M. Eskandari Z. Ramezani S. Pirzeh R. Dadashi M. (2021). The effect of quantitative electroencephalography-based neurofeedback therapy on anxiety, depression, and emotion regulation in people with generalized anxiety disorder. Basic Clin. Neurosci. 12, 281–290. doi: 10.32598/BCN.12.2.2378.1 34925724 PMC8672673

[ref2] Alexopoulos D. S. Foudoulaki E. (2002). Construct validity of the Piers-Harris Children’s self-concept scale. Psychol. Rep. 91, 827–838. doi: 10.2466/pr0.2002.91.3.827, PMID: 12530730

[ref3] Altarac M. Saroha E. (2007). Lifetime prevalence of learning disability among US children. Pediatrics 119, Suppl 1, S77–S83. doi: 10.1542/peds.2006-2089L 17272589

[ref5] American Psychiatric Association (2000). Diagnostic and statistical manual of mental disorders, (4th ed., text rev.).

[ref4] American Psychiatric Association (2013). Diagnostic and statistical manual of mental disorders (5th ed.). doi: 10.1176/appi.books.9780890425596

[ref6] Angelakis E. Stathopoulou S. Frymiare J. L. Green D. L. Lubar J. F. Kounios J.. (2007). EEG neurofeedback: a brief overview and an example of peak alpha frequency training for cognitive enhancement in the elderly. Clin. Neuropsychol. 21, 110–129. doi: 10.1080/13854040600744839 17366280

[ref7] American Psychiatric Association. (2022). Diagnostic and statistical manual of mental disorders (5th ed., text rev.). doi: 10.1176/appi.books.9780890425787

[ref8] World Medical Association. (2013). World medical association declaration of Helsinki: ethical principles for medical research involving human subjects. JAMA 310, 2191–2194. doi: 10.1001/jama.2013.281053 24141714

[ref9] Baumeister R. F. Campbell J. D. Krueger J. I. Vohs K. D. (2003). Does high self-esteem cause better performance, interpersonal success, happiness, or healthier lifestyles? Psychol. Sci. Public Interes. 4, 1–44. doi: 10.1111/1529-1006.01431 26151640

[ref10] Becerra J. Fernández T. Harmony T. Caballero M. I. Garcia F. Fernández-Bouzas A.. (2006). Follow-up study of learning-disabled children treated with neurofeedback or placebo. Clin. EEG Neurosci. 37, 198–203. doi: 10.1177/155005940603700307 16929704

[ref11] Block C. C. (1993). Strategy instruction in a literature-based reading program. Elem. Sch. J. 94, 139–151. doi: 10.1086/461756

[ref12] Bosch-Bayard J. Aubert-Vazquez E. Brown S. T. Rogers C. Kiar G. Glatard T.. (2020a). A quantitative EEG toolbox for the MNI neuroinformatics ecosystem: normative SPM of EEG source spectra. Front. Neuroinform. 14:33. doi: 10.3389/fninf.2020.00033, PMID: 32848689 PMC7427620

[ref13] Bosch-Bayard J. Galan L. Aubert Vazquez E. Virues Alba T. Valdes-Sosa P. A. (2020). Resting state healthy EEG: the first wave of the cuban normative database. Front. Neurosci. 14:555119 doi: 10.3389/fnins.2020.555119, PMID: 33335467 PMC7736237

[ref14] Bosch-Bayard J. Valdés-Sosa P. Virues-Alba T. Aubert-Vázquez E. John E. R. Harmony T.. (2001). 3D statistical parametric mapping of EEG source spectra by means of variable resolution electromagnetic tomography (VARETA). Clin. EEG Neurosci. 32, 47–61. doi: 10.1177/155005940103200203, PMID: 11360721

[ref15] Breteler M. H. M. Arns M. Peters S. Giepmans I. Verhoeven L. (2010). Improvements in spelling after QEEG-based neurofeedback in dyslexia: a randomized controlled treatment study. Appl. Psychophysiol. Biofeedback 35, 5–11. doi: 10.1007/s10484-009-9105-2, PMID: 19711183 PMC2837193

[ref16] Budzynski T. H. Budzynski H. K. Evans J. R. Abarbanel A. (2009a). “Introduction,” in Introduction to quantitative EEG and neurofeedback (Second Edition), eds. Budzynski T. H. Budzynski H. K. Evans J. R. Abarbanel A. (San Diego: Academic Press), xxi–xxii. doi: 10.1016/B978-0-12-374534-7.00020-4

[ref17] Carroll J. M. Maughan B. Goodman R. Meltzer H. (2005). Literacy difficulties and psychiatric disorders: evidence for comorbidity. J. Child Psychol. Psychiatry Allied Discip. 46, 524–532. doi: 10.1111/j.1469-7610.2004.00366.x, PMID: 15845132

[ref18] Chabot R. J. (2001). The clinical role of computerized EEG in the evaluation and treatment of learning and attention disorders in children and adolescents. J. Neuropsychiatr. 13, 171–186. doi: 10.1176/appi.neuropsych.13.2.171, PMID: 11449024

[ref19] Chen C. Xiao X. Belkacem A. N. Lu L. Wang X. Yi W.. (2021). Efficacy evaluation of neurofeedback-based anxiety relief. Front. Neurosci. 15:758068. doi: 10.3389/fnins.2021.758068, PMID: 34776855 PMC8581142

[ref20] Choi S. W. Chi S. E. Chung S. Y. Kim J. W. Ahn C. Y. Kim H. T. (2010). Is alpha wave neurofeedback effective with randomized clinical trials in depression? A pilot study. Neuropsychobiology 63, 43–51. doi: 10.1159/000322290, PMID: 21063132

[ref21] Choi Y. Choi S. H. Yun J. Y. Lim J. A. Kwon Y. Lee H. Y.. (2019). The relationship between levels of self-esteem and the development of depression in young adults with mild depressive symptoms. Medicine (Baltimore) 98:e17518. doi: 10.1097/MD.0000000000017518, PMID: 31626112 PMC6824750

[ref22] Colloca L. Miller F. G. (2011). How placebo responses are formed: a learning perspective. Philos. Trans. R. Soc. B Biol. Sci. 366, 1859–1869. doi: 10.1098/rstb.2010.0398, PMID: 21576143 PMC3130403

[ref23] Cooley E. J. Ayres R. R. (1988). Self-concept and success-failure attributions of nonhandicapped students and students with learning disabilities. J. Learn. Disabil. 21, 174–178. doi: 10.1177/002221948802100310, PMID: 3351387

[ref24] Daniel S. S. Walsh A. K. Goldston D. B. Arnold E. M. Reboussin B. A. Wood F. B. (2006). Suicidality, school dropout, and reading problems among adolescents. J. Learn. Disabil. 39, 507–514. doi: 10.1177/00222194060390060301, PMID: 17165618

[ref25] Egner T. Sterman M. B. (2006). Neurofeedback treatment of epilepsy: from basic rationale to practical application. Expert. Rev. Neurother. 6, 247–257. doi: 10.1586/14737175.6.2.247, PMID: 16466304

[ref26] Epstein S. (1973). The self-concept revisited. Or a theory of a theory. Am. Psychol. 28, 404–416. doi: 10.1037/h0034679, PMID: 4703058

[ref27] Fernández T. Bosch-Bayard J. Harmony T. Caballero M. I. Díaz-Comas L. Galán L.. (2016). Neurofeedback in learning disabled children: visual versus auditory reinforcement. Appl. Psychophysiol. Biofeedback 41, 27–37. doi: 10.1007/s10484-015-9309-6, PMID: 26294269

[ref28] Fernández T. Harmony T. Fernández-Bouzas A. Díaz-Comas L. Prado-Alcalá R. A. Valdés-Sosa P.. (2007). Changes in EEG current sources induced by neurofeedback in learning disabled children. An exploratory study. Appl. Psychophysiol. Biofeedback 32, 169–183. doi: 10.1007/s10484-007-9044-8, PMID: 17978869

[ref29] Fernández T. Harmony T. Fernández-Bouzas A. Silva J. Herrera W. Santiago-Rodríguez E.. (2002). Sources of EEG activity in learning disabled children. Clin. EEG Neurosci. 33, 160–164. doi: 10.1177/155005940203300405, PMID: 12449846

[ref30] Fernández T. Herrera W. Harmony T. Díaz-Comas L. Santiago E. Sánchez L.. (2003). EEG and behavioral changes following neurofeedback treatment in learning disabled children. Clin. EEG Neurosci. 34, 145–152. doi: 10.1177/155005940303400308, PMID: 14521276

[ref31] Flahive M. H. W. Chuang Y. C. Li C. M. (2015). The multimedia piers-Harris children’s self-concept scale 2: its psychometric properties, equivalence with the paper-and-pencil version, and respondent preferences. PLoS One 10:e0135386. doi: 10.1371/journal.pone.0135386, PMID: 26252499 PMC4529174

[ref32] Flores-Gallegos R. Rodríguez-Leis P. Fernández T. (2022). Effects of a virtual reality training program on visual attention and motor performance in children with reading learning disability. Int. J. Child Comput. Interact. 32:100394. doi: 10.1016/j.ijcci.2021.100394

[ref33] Fonseca L. C. Tedrus G. M. A. S. Chiodi M. G. Cerqueira J. N. Tonelotto J. M. F. (2006). Quantitative EEG in children with learning disabilities: analysis of band power. Arq. Neuropsiquiatr. 64, 376–381. doi: 10.1590/S0004-282X2006000300005, PMID: 16917604

[ref34] Francis D. A. Caruana N. Hudson J. L. McArthur G. M. (2019). The association between poor reading and internalising problems: a systematic review and meta-analysis. Clin. Psychol. Rev. 67, 45–60. doi: 10.1016/j.cpr.2018.09.002, PMID: 30528985

[ref35] Gans A. M. Kenny M. C. Ghany D. L. (2003). Comparing the self-concept of students with and without learning disabilities. J. Learn. Disabil. 36, 287–295. doi: 10.1177/002221940303600307 15515648

[ref36] Geuter S. Koban L. Wager T. D. (2017). The cognitive neuroscience of placebo effects: concepts, predictions, and physiology. Annu. Rev. Neurosci. 40, 167–188. doi: 10.1146/annurev-neuro-072116-031132, PMID: 28399689

[ref37] González-Pienda J. A. Núñez J. C. González-Pumariega S. Álvarez L. Roces C. García M.. (2000). Autoconcepto, proceso de atribución causal y metas académicas en niños con y sin dificultades de aprendizaje. Psicothema 12, 548–556.

[ref38] Gruzelier J. H. (2014). EEG-neurofeedback for optimising performance. I: a review of cognitive and affective outcome in healthy participants. Neurosci. Biobehav. Rev. 44, 124–141. doi: 10.1016/j.neubiorev.2013.09.015, PMID: 24125857

[ref39] Hammond D. C. (2006). Journal of neurotherapy: investigations in neuromodulation, neurofeedback and applied neuroscience. J. Neurother. 10, 25–36. doi: 10.1300/J184v03n01

[ref40] Hards E. Ellis J. Fisk J. Reynolds S. (2020). Negative view of the self and symptoms of depression in adolescents. J. Affect. Disord. 262, 143–148. doi: 10.1016/j.jad.2019.11.012, PMID: 31733458

[ref41] Holcomb P. J. Ackerman P. T. Dykman R. A. (1986). Auditory event-related potentials in attention and reading disabled boys. Int. J. Psychophysiol. 3, 263–273. doi: 10.1016/0167-8760(86)90035-8, PMID: 3700187

[ref42] Huang C. (2011). Self-concept and academic achievement: a meta-analysis of longitudinal relations. J. Sch. Psychol. 49, 505–528. doi: 10.1016/j.jsp.2011.07.001, PMID: 21930007

[ref43] Huang A. Sun M. Zhang X. Lin Y. Lin X. Wu K.. (2021). Self-concept in primary school student with dyslexia: the relationship to parental rearing styles. Int. J. Environ. Res. Public Health 18:9718. doi: 10.3390/ijerph18189718, PMID: 34574639 PMC8466928

[ref44] Huang C. Zhang H. Huang J. Duan C. Kim J. J. Ferrari M.. (2020). Stronger resting-state neural oscillations associated with wiser advising from the 2nd- but not the 3rd-person perspective. Sci. Rep. 10:12677. doi: 10.1038/s41598-020-69507-9, PMID: 32728108 PMC7391636

[ref45] Kutner M. Greenberg E. Jin Y. Paulsen C. (2006). The health literacy of America’s adults: results from the 2003 national assessment of adult literacy. (NCES 2006–483). U.S. Department of Education. Washington, DC: National Center for Education Statistics

[ref46] Lagae L. (2008). Learning disabilities: definitions, epidemiology, diagnosis, and intervention strategies. Pediatr. Clin. N. Am. 55, 1259–1268. doi: 10.1016/j.pcl.2008.08.001, PMID: 19041456

[ref47] Lahane S. Shah H. Nagarale V. Kamath R. (2013). Comparison of self-esteem and maternal attitude between children with learning disability and unaffected siblings. Indian J. Pediatr. 80, 745–749. doi: 10.1007/s12098-012-0915-5, PMID: 23180409

[ref48] Lewis K. Kuhfeld M. Ruzek E. McEachin A. (2021). Learning during COVID-19: reading and math achievement in the 2020–21 school year. NWEA. Available at: https://www.nwea.org/uploads/2021/07/Learning-during-COVID-19-Reading-and-math-achievement-in-the-2020-2021-school-year.research-brief-1.pdf

[ref49] López-Pinar C. Martínez-Sanchís S. Carbonell-Vayá E. Sánchez-Meca J. Fenollar-Cortés J. (2020). Efficacy of nonpharmacological treatments on comorbid internalizing symptoms of adults with attention-deficit/hyperactivity disorder: a Meta-analytic review. J. Atten. Disord. 24, 456–478. doi: 10.1177/1087054719855685, PMID: 31189374

[ref50] Lubar J. F. Swartwood M. O. Swartwood J. N. O’Donnell P. H. (1995). Evaluation of the effectiveness of EEG neurofeedback training for ADHD in a clinical setting as measured by changes in T.O.V.a. scores, behavioral ratings, and WISC-R performance. Biofeedback Self Regul. 20, 83–99. doi: 10.1007/BF01712768, PMID: 7786929

[ref51] MacMahon J. R. Gross R. T. (1987). Physical and psychological effects of aerobic exercise in boys with learning disabilities. J. Dev. Behav. Pediatr. 8, 274–277. doi: 10.1097/00004703-198710000-00006 3680538

[ref52] Marsh H. W. (1990). A multidimensional, hierarchical model of self-concept: theoretical and empirical justification. Educ. Psychol. Rev. 2, 77–172. doi: 10.1007/BF01322177

[ref53] Martínez-Briones B. J. Bosch-Bayard J. Biscay-Lirio R. J. Silva-Pereyra J. Albarrán-Cárdenas L. Fernández T. (2021). Effects of neurofeedback on the working memory of children with learning disorders-an EEG power-spectrum analysis. Brain Sci. 11: 957. doi: 10.3390/brainsci11070957, PMID: 34356191 PMC8303215

[ref54] Matute E. Inozemtseva O. Gonzalez A. L. Chamorro Y. (2014). La Evaluación Neuropsicológica Infantil (ENI): Historia y fundamentos teóricos de su validación, un acercamiento práctico a su uso y valor diagnóstico. Available at: http://revistaneurociencias.com/index.php/RNNN/article/view/44

[ref55] McArthur G. Castles A. Kohnen S. Banales E. (2016). Low self-concept in poor readers: prevalence, heterogeneity, and risk. PeerJ 4:e2669. doi: 10.7717/peerj.2669, PMID: 27867764 PMC5111895

[ref56] McArthur G. M. Filardi N. Francis D. A. Boyes M. E. Badcock N. A. (2020). Self-concept in poor readers: a systematic review and meta-analysis. PeerJ 8:e8772. doi: 10.7717/peerj.8772, PMID: 32211239 PMC7081778

[ref57] Mennella R. Patron E. Palomba D. (2017). Frontal alpha asymmetry neurofeedback for the reduction of negative affect and anxiety. Behav. Res. Ther. 92, 32–40. doi: 10.1016/j.brat.2017.02.002, PMID: 28236680

[ref58] Morales-Quezada L. Martinez D. El-Hagrassy M. M. Kaptchuk T. J. Sterman M. B. Yeh G. Y. (2019). Neurofeedback impacts cognition and quality of life in pediatric focal epilepsy: an exploratory randomized double-blinded sham-controlled trial. Epilepsy Behav. 101:106570. doi: 10.1016/j.yebeh.2019.106570, PMID: 31707107 PMC7203763

[ref59] Musetti A. Eboli G. Cavallini F. Corsano P. (2019). Social relationships, self-esteem, and loneliness in adolescents with learning disabilities. Clin. Neuropsychiatry 16, 165–172. PMID: 34908952 PMC8650192

[ref60] National Reading Panel (2000). Teaching children to read: an evidence-based assessment of the scientific research literature on reading and its implications for reading instruction. Available at: http://www.nichd.nih.gov/publications/nrp/upload/smallbook_pdf.pdf

[ref61] Nazari M. A. Mosanezhad E. Hashemi T. Jahan A. (2012). The effectiveness of neurofeedback training on EEG coherence and neuropsychological functions in children with reading disability. Clin. EEG Neurosci. 43, 315–322. doi: 10.1177/1550059412451880, PMID: 23185091

[ref62] Nelson J. M. Gregg N. (2012). Depression and anxiety among transitioning adolescents and college students with ADHD, dyslexia, or comorbid ADHD/dyslexia. J. Atten. Disord. 16, 244–254. doi: 10.1177/1087054710385783, PMID: 20978271

[ref63] Núñez Pérez J. C. González Pumariega S. González Pienda J. A. (1995). Autoconcepto en niños con y sin dificultades de aprendizaje. Psicothema 7, 587–604.

[ref64] Orgilés M. Morales A. Delvecchio E. Mazzeschi C. Espada J. P. (2020). Immediate psychological effects of the COVID-19 quarantine in youth from Italy and Spain. Front. Psychol. 11:579038. doi: 10.3389/fpsyg.2020.579038, PMID: 33240167 PMC7677301

[ref65] Parker R. I. Vannest K. (2009). An improved effect size for single-case research: nonoverlap of all pairs. Behav. Ther. 40, 357–367. doi: 10.1016/j.beth.2008.10.006, PMID: 19892081

[ref66] Parker R. I. Vannest K. J. Davis J. L. (2011). Effect size in single-case research: a review of nine nonoverlap techniques. Behav. Modif. 35, 303–322. doi: 10.1177/0145445511399147, PMID: 21411481

[ref67] Pascual-Marqui R. D. Lehmann D. Koukkou M. Kochi K. Anderer P. Saletu B.. (2011). Assessing interactions in the brain with exact low-resolution electromagnetic tomography. Philos. Trans. R. Soc. A Math. Phys. Eng. Sci. 369, 3768–3784. doi: 10.1098/rsta.2011.0081 21893527

[ref68] Piers E. V Herzberg D. S. (2002). Piers-Harris Children’s self-concept scale: manual. Western Psychological Corporation. Available at: https://books.google.com.mx/books?id=-S12zQEACAAJ

[ref69] Qiu Y. Mao Z. Yun D. (2022). Can the add-on placebo effect augment the physical and mental health outcomes of exercise? A meta-analysis. Appl. Psychol. Heal. Well-Being 14, 483–498. doi: 10.1111/aphw.12315, PMID: 34749434

[ref70] Review of the Piers-Harris Children’s Self-Concept Scale 2nd Edition (2011). Community-university Partnersh. Available at: (https://www.ualberta.ca/community-university-partnership/media-library/community-university-partnership/resources/tools-assessment/piers-harris-2may-2012.pdf).

[ref71] Schönenberg M. Weingärtner A. L. Weimer K. Scheeff J. (2021). Believing is achieving - on the role of treatment expectation in neurofeedback applications. Prog. Neuro-Psychopharmacology Biol. Psychiatry 105:110129. doi: 10.1016/j.pnpbp.2020.110129, PMID: 33031860

[ref72] Shaywitz S. E. Fletcher J. M. Holahan J. M. Shneider A. E. Marchione K. E. Stuebing K. K.. (1999). Persistence of dyslexia: the Connecticut longitudinal study at adolescence. Pediatrics 104, 1351–1359. doi: 10.1542/peds.104.6.1351, PMID: 10585988

[ref73] Shaywitz S. Shaywitz J. (2020). Overcoming dyslexia: completely revised and updated. 2nd New York: Alfred A. Knopf

[ref74] Silva-Pereyra J. Rivera-Gaxiola M. Fernández T. Díaz-Comas L. Harmony T. Fernández-Bouzas A.. (2003). Are poor readers semantically challenged? An event-related brain potential assessment. Int. J. Psychophysiol. 49, 187–199. doi: 10.1016/S0167-8760(03)00116-8, PMID: 14507438

[ref75] Simkin D. R. Arnold L. E. Lubar J. (2016). Neurofeedback in attention-deficit/hyperactivity disorder: evaluation difficulties. J. Am. Acad. Child Adolesc. Psychiatry 55, 1090–1091. doi: 10.1016/j.jaac.2016.09.494, PMID: 27871644

[ref76] Simkin D. R. Thatcher R. W. Lubar J. (2014). Quantitative EEG and neurofeedback in children and adolescents. Anxiety disorders, depressive disorders, comorbid addiction and attention-deficit/hyperactivity disorder, and brain injury. Child Adolesc. Psychiatr. Clin. N. Am. 23, 427–464. doi: 10.1016/j.chc.2014.03.001, PMID: 24975621

[ref77] Sitaram R. Ros T. Stoeckel L. Haller S. Scharnowski F. Lewis-Peacock J.. (2017). Closed-loop brain training: the science of neurofeedback. Nat. Rev. Neurosci. 18, 86–100. doi: 10.1038/nrn.2016.164, PMID: 28003656

[ref78] Smith D. S. Nagle R. J. (1995). Self-perceptions and social comparisons among children with LD. J. Learn. Disabil. 28, 364–371. doi: 10.1177/002221949502800607, PMID: 7622968

[ref79] Snowling M. J. Muter V. Carroll J. (2007). Children at family risk of dyslexia: a follow-up in early adolescence. J. Child Psychol. Psychiatry Allied Discip. 48, 609–618. doi: 10.1111/j.1469-7610.2006.01725.x, PMID: 17537077

[ref80] Sowislo J. F. Orth U. (2013). Does low self-esteem predict depression and anxiety? A meta-analysis of longitudinal studies. Psychol. Bull. 139, 213–240. doi: 10.1037/a0028931, PMID: 22730921

[ref81] Sterman M. B. Egner T. (2006). Foundation and practice of neurofeedback for the treatment of epilepsy. Appl. Psychophysiol. Biofeedback 31, 21–35. doi: 10.1007/s10484-006-9002-x, PMID: 16614940

[ref82] Swanson H. L. Hoskyn M. (1998). Experimental intervention research on students with learning disabilities: a meta-analysis of treatment outcomes. Rev. Educ. Res. 68, 277–321. doi: 10.3102/00346543068003277

[ref83] Terras M. M. Thompson L. C. Minnis H. (2009). Dyslexia and psycho-social functioning: an exploratory study of the role of self-esteem and understanding. Dyslexia 15, 304–327. doi: 10.1002/dys.386, PMID: 19384920

[ref84] Thibault R. T. Raz A. (2016). When can neurofeedback join the clinical armamentarium? Lancet Psychiatry 3, 497–498. doi: 10.1016/S2215-0366(16)30040-2, PMID: 27262039

[ref85] Thornton K. E. (2018). Perspectives on placebo: the psychology of neurofeedback. NeuroRegulation 5, 137–149. doi: 10.15540/nr.5.4.137

[ref86] Tobia V. Riva P. Caprin C. (2017). Who are the children Most vulnerable to social exclusion? The moderating role of self-esteem, popularity, and nonverbal intelligence on cognitive performance following social exclusion. J. Abnorm. Child Psychol. 45, 789–801. doi: 10.1007/s10802-016-0191-3, PMID: 27457234

[ref87] Vannest K. J. Parker R. I. Gonen O. Adiguzel T. (2016). Single case research: Web-based calculators for SCR analysis, Version 2.0. College Station, TX: Texas A&M University

[ref88] Wechsler D. (2010). WISC-IV, Escala de inteligencia de wechsler para Niños-IV. Manual técnico y de interpretación. TEA Ediciones, S.A. Available at: https://books.google.com.mx/books?id=AH8rPQAACAAJ.

[ref89] Willcutt E. G. Pennington B. F. (2000). Psychiatric comorbidity in children and adolescents with reading disability. J. Child Psychol. Psychiatry Allied Discip. 41, 1039–1048. doi: 10.1017/S0021963099006368 11099120

[ref90] Xie X. Xue Q. Zhou Y. Zhu K. Liu Q. Zhang J.. (2020). Mental health status among children in home confinement during the coronavirus disease 2019 outbreak in Hubei Province. China JAMA Pediatr. 174, 898–900. doi: 10.1001/jamapediatrics.2020.1619, PMID: 32329784 PMC7182958

[ref91] Zeleke S. (2004). Self-concepts of students with learning disabilities and their normally achieving peers: a review. Eur. J. Spec. Needs Educ. 19, 145–170. doi: 10.1080/08856250410001678469

