# Supplementary material for: Effects of neurofeedback on the self-concept of children with learning disorders
Source: Front Psychol. 2023 May 15;14:1167961. doi: 10.3389/fpsyg.2023.1167961 (PMC10225657; doi:10.3389/fpsyg.2023.1167961)
Supplement: Supplementary file 1 [file Table_1.DOC]

Supplementary Material

# Effects of Neurofeedback on the Self-Concept of Children with Learning Disorders

Benito Javier Martínez-Briones^†^, Rodrigo Flores-Gallegos^†^, Sonia Y Cárdenas, Bertha Elena Barrera-Díaz, Thalía Fernández^*^, Juan Silva-Pereyra^*^

^†^These authors share first authorship

*These authors share senior authorship

*Correspondence:

Thalía Fernández^1^; Juan Silva-Pereyra^3^

[thaliafh@yahoo.com.mx](mailto:thaliafh@yahoo.com.mx); [jsilvapereyra@gmail.com](mailto:jsilvapereyra@gmail.com)

# Supplementary Tables

**Table S1.**Mean difference (pre vs. post) between Neurofeedback (NFB) and Control (CTRL) groups for reading, writing, and mathematics percentile averages.

| **Variable** | **NFB Post-Pre**  **Mean (SD)** | **CTRL Post-Pre Mean (SD)** | **t** | **p** | **Cohen d** |
| --- | --- | --- | --- | --- | --- |
| Reading | 11.83 (15.30) | 8.45 (19.66) | 0.58 | 0.62 | 0.17 |
| Writing | 2.36(14.80) | 1.69(15.80) | 0.13 | 0.80 | 0.03 |
| Mathematics | 7.74(15.75) | -8.78(18.88) | 2.860 | 0.01 | 0.80 |

**Table S2.**Reading, writing, and mathematics percentiles after training for the Neurofeedback group.

| **Variable** | **Mean pre (SD)** | **Mean post (SD)** | **t** | **p** |  | **Cohen d** |
| --- | --- | --- | --- | --- | --- | --- |
| Reading | 29.76(20.24) | 41.59(19.25) | 3.46 | 0.01 |  | 0.60 |
| Writing | 38.44(20.59) | 40.81(16.13) | 0.73 | 0.51 |  | 0.12 |
| Mathematics | 33.05(21.78) | 40.79(19.47) | 2.20 | 0.06 |  | 0.40 |

**Table S3.**Reading, writing, and mathematics percentiles after training for the Control group.

| **Variable** | **Mean pre (SD)** | **Mean post (SD)** | **t** | **p** | **Cohen d** |
| --- | --- | --- | --- | --- | --- |
| Reading | 21.16(20.48) | 29.61(21.59) | 1.61 | 0.19 | 0.40 |
| Writing | 28.82(17.80) | 30.52(23.74) | 0.40 | 0.69 | 0.08 |
| Mathematics | 41.01(21.78) | 32.23(22.90) | -1.74 | 0.13 | 0.39 |

**Table S4.**Self-concept dimensions after training for the Neurofeedback group.

| **Variable** | **Mean pre (SD)** | **Mean post (SD)** | **t** | **p** | **Cohen d** |
| --- | --- | --- | --- | --- | --- |
| Behavior | 11.85(3.88) | 13.30(3.80) | 1.98 | 0.13 | 0.38 |
| Intelligence | 10.55(2.26) | 11.40(2.66) | 1.44 | 0.33 | 0.34 |
| Physical | 9.65(2.18) | 11.00(2.96) | 3.09 | 0.01 | 0.49 |
| No anxiety | 5.70(2.62) | 7.30(2.66) | 2.86 | 0.02 | 0.61 |
| Popularity | 7.75(2.40) | 8.95(1.96) | 2.73 | 0.03 | 0.54 |
| Happiness | 7.65(2.80) | 9.10(1.68) | 2.55 | 0.04 | 0.60 |

**Table S5.** Global self-concept and self-concept dimensions after training for the Control group.

| **Variable** | **Mean pre (SD)** | **Mean post (SD)** | **t** | **p** | **Cohen d** |
| --- | --- | --- | --- | --- | --- |
| Behavior | 14.71 (2.95) | 15.21(2.89) | 0.57 | 0.68 | 0.17 |
| Intelligence | 12.07(1.77) | 11.57(3.03) | -0.78 | 0.99 | 0.18 |
| Physical | 10.43(1.867) | 10.36(1.95) | -0.10 | 0.90 | 0.04 |
| No anxiety | 5.29(2.30) | 6.21(2.04) | 1.21 | 0.39 | 0.43 |
| Popularity | 8.71(1.77) | 9.21(2.46) | 0.89 | 0.55 | 0.23 |
| Happiness | 8.64(1.78) | 9.36(2.31) | 1.14 | 0.43 | 0.34 |
